# Hysteresis Behavior of the Donor–Acceptor-Type Ambipolar Semiconductor for Non-Volatile Memory Applications

**DOI:** 10.3390/mi12030301

**Published:** 2021-03-12

**Authors:** Young Jin Choi, Jihyun Kim, Min Je Kim, Hwa Sook Ryu, Han Young Woo, Jeong Ho Cho, Joohoon Kang

**Affiliations:** 1 Department of Chemical and Biomolecular Engineering, Yonsei University, Seoul 03722, Korea; cyjhe123@gmail.com (Y.J.C.); rlaalswp910@gmail.com (M.J.K.); jhcho94@yonsei.ac.kr (J.H.C.); 2 School of Advanced Materials Science and Engineering, Sungkyunkwan University (SKKU), Suwon 16419, Korea; jeeh8959@g.skku.edu; 3 Department of Chemistry, Korea University, Seoul 02841, Korea; rhs0104@naver.com (H.S.R.); hywoo@korea.ac.kr (H.Y.W.)

**Keywords:** organic semiconductors, donor–acceptor-type molecules, ambipolar field-effect transistors, non-volatile memory, charge trapping

## Abstract

Donor–acceptor-type organic semiconductor molecules are of great interest for potential organic field-effect transistor applications with ambipolar characteristics and non-volatile memory applications. Here, we synthesized an organic semiconductor, PDPPT-TT, and directly utilized it in both field-effect transistor and non-volatile memory applications. As-synthesized PDPPT-TT was simply spin-coated on a substrate for the device fabrications. The PDPPT-TT based field-effect transistor showed ambipolar electrical transfer characteristics. Furthermore, a gold nanoparticle-embedded dielectric layer was used as a charge trapping layer for the non-volatile memory device applications. The non-volatile memory device showed clear memory window formation as applied gate voltage increases, and electrical stability was evaluated by performing retention and cycling tests. In summary, we demonstrate that a donor–acceptor-type organic semiconductor molecule shows great potential for ambipolar field-effect transistors and non-volatile memory device applications as an important class of materials.

## 1. Introduction

Organic molecule-based electronic device applications are of great interest with a wide range of materials for selection and their great potential for use in transparent, flexible device applications in large scales and memory device applications due to their solution processability in large areas with high spatial uniformity on any arbitrary substrates [1,2,3,4,5,6,7]. Among the material lists, semiconducting organic molecules have been more extensively investigated to apply as a channel material for organic field-effect transistors (OFETs). A general structure of the semiconducting organic molecules consists of electron-rich donor or electron-deficient acceptor blocks as a donor–acceptor-type semiconductor (e.g., thiophenes [8] and selenophenes [9] are in the type of electron-rich donors, and isoindigos [10,11], benzothiadiazoles [12], and naphthalenedicarboximide [13] are in the type of electron-deficient acceptors, respectively).

As one of the electron-deficient acceptor blocks, diketopyrrolopyrrole (DPP)-based polymers have been given more attention due to their relatively good electrical properties, including charge carrier mobilities compatible to 1 cm^2^/Vs [14,15,16]. The high carrier mobilities mainly originate from the strong π–π interactions between DPP moieties [17,18]. Additionally, such DPP-based semiconductors often exhibit ambipolar transfer characteristics due to their highest occupied molecular orbital (HOMO) and lowest unoccupied molecular orbital (LUMO) levels being close to the Fermi level of OFET electrodes. Furthermore, DPP derivatives such as DPP-thieno(3,2-b)thiophene (PDPPT) have also been extensively studied due to their excellent solution processability and improved charge carrier mobilities for OFET applications [15,19,20].

In this paper, we report the synthesis of a DPP-based organic semiconductor, poly(2,5-(2-decyltetradecyl)-3,6-diketopyrrolopyrrole-alt-5,5-(2,5-di(thien-2-yl)thieno(3,2-b)thiophene)) (PDPPT-TT). The electrical characterizations of the PDPPT-TT based transistors were evaluated by the fabrication of OFETs with a spin-coated PDPPT-TT layer, which showed the ambipolar electrical characteristics with a hole mobility of 0.037 cm^2^/Vs. Additionally, this OFET exhibits the evolution of hysteresis loop window as the applied gate voltage sweep range increases for the potential in non-volatile memory device applications. However, the memory device performance was not ideal with the conventional OFET structure due to the shallow trap-induced short retention time. To improve the non-volatile memory device performance, including the stable retention time and cycling of programed/erased states, a gold nanoparticle (AuNP)-embedded dielectric layer was introduced as an efficient charge-trapping layer. The resulting AuNP-embedded dielectric layer incorporated PDPPT-TT-based OFETs, exhibiting optimized non-volatile memory performance with high stability.

## 2. Materials and Methods

### 2.1. Synthesis of PDPPT-TT

**Figure 1:**
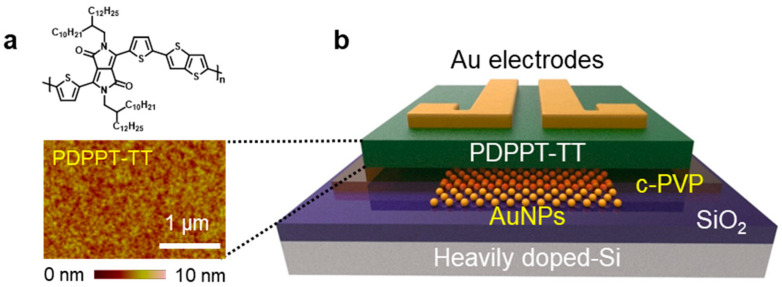
(**a**) Molecular structure of as-synthesized PDPPT-TT and atomic force microscopic image of as-coated PDPPT-TT layer; (**b**) schematic of the PDPPT-TT-based thin-film transistor containing c-PVP/AuNPs layers for non-volatile memory applications.

As a semiconducting channel material, we synthesized poly (2,5-(2-decyltetradecyl)-3,6-diketopyrrolopyrrole-alt-5,5-(2,5-di(thien-2-yl)thieno(3,2-b)thiophene)), or PDPPT-TT in short. In Figure 1a, the molecular structure of PDPPT-TT is illustrated with a highly planar well-conjugated polymer backbone. This polymeric semiconductor was synthesized followed by the sequence: (i) the synthesis of 3,6-Di(thiophen-2-yl)pyrrolo[3,4-c]pyrrole-1,4 (2H,5H)-dione (DBT-H) and 3,6-bis (5-bromothiophen-2-yl)-2,5-bis (2-decyltetradecyl) pyrrolo(3,4-c)pyrrole-1,4(2H,5H)-dione (M-24) was followed from previous reports [15,19,20]; (ii) 0.1613 g of dibromo-DPP M-24, 0.07 g of 2,5-bis(trimethylstannyl)thieno(3,2-b)thiophene, and 3.7 mg of tri(o-totyl)phosphine were mixed in a 50 mL dry flask. The dry flask was purged with ultra-high purity (UHP) Ar gas three times to remove the residual O_2_. In the mixture, 13 mL of anhydrous chlorobenzene and 2.8 mg of tris-(dibenzylideneacetone)dipalladium(0) (Pd_2_(dba)_3_) were added and the mixture was stirred at 130 °C for 3 d in an Ar atmosphere. Then, the mixture was naturally cooled to room temperature and 0.5 mL of 2-bromothiophene was added. The mixture was heated to 130 °C for 2 h with stirring. Once the reaction was stopped, the mixture was cooled to room temperature again, and the resulting polymers were purified and filtered via precipitation in methanol. Lastly, ~150 mg of PDPPT-TT was obtained after Soxhlet extraction with acetone and hexane to remove the residual organic impurities and oligomers having low molecular weight, subsequent extraction with chloroform, and drying under high vacuum [14].

### 2.2. PDPPT-TT-Based Transistor Fabrication

A heavily doped 300 nm-thick SiO_2_/Si substrate was cleaned by dipping in a piranha solution for 30 min at 100 °C and rinsed with deionized water. To avoid the charge trapping at the interface between the SiO_2_ substrate and the PDPPT-TT channel due to the presence of the hydroxyl group, a self-assembled monolayer of octadecyltrichlorosilane (ODTS, purchased from Gelest Inc., Morrisville, PA, USA) was formed on the SiO_2_ surface. After the ODTS treatment, the semiconducting PDPPT-TT layer was formed on the substrate by spin-coating of a 0.2 wt.% solution in chloroform. The surface roughness of the PDPPT-TT layer was characterized by using atomic force microscopy (AFM), as shown in Figure 1a. The spin-coated PDPPT-TT layer was dried for 24 h and annealed at 200 °C for 30 min in a vacuum chamber. Lastly, source and drain electrodes were formed on the PDPPT-TT layer by a thermal deposition of 50 nm-thick Au. To demonstrate non-volatile memory performance, gold nanoparticles (AuNPs) were introduced between the SiO_2_ and the PDPPT-TT layer. A 1 nm-thick Au layer was thermally deposited and subsequently annealed at 100 °C for 10 min to form the AuNPs. Subsequently, a cross-linked poly (4-vinylphenol) (c-PVP) was formed by spin-coating PVP with a cross-linking agent, poly (melamine-co-formaldehyde) (PMF) in a weight ratio of 2:1, and the entire sample was annealed at 150 °C for 1 h. The fabricated devices were measured in a vacuum probe station (~10^3^ Torr). The device structure is illustrated in Figure 1b.

## 3. Results and Discussion

### 3.1. Electrical Properties of PDPPT-TT

**Figure 2:**
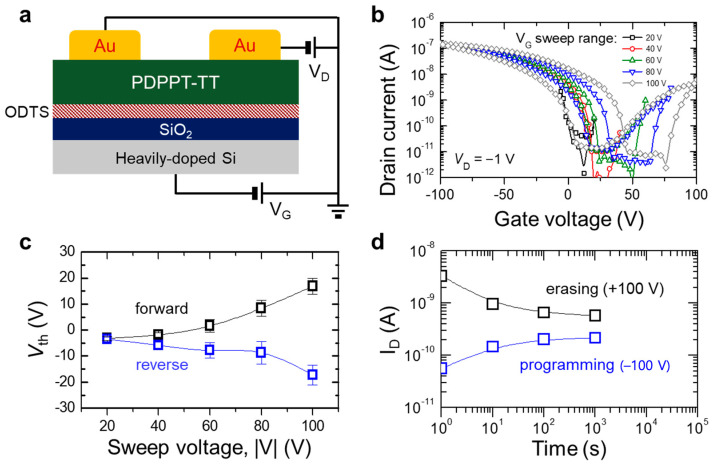
(**a**) Schematic of a PDPPT-TT-based transistor; (**b**) transfer characteristics of the device exhibiting ambipolar behavior; (**c**) summarized plot of the memory windows defined as the threshold voltages of forward and reverse biases; (**d**) retention time of the device with application of gate voltage of ±100 V.

The electrical properties of the semiconducting PDPPT-TT were characterized without incorporation of the c-PVP/AuNP layer (i.e., the PDPPT-TT layer prepared on the ODTS-treated SiO_2_/Si substrate) as shown in Figure 2a. Figure 2b shows transfer characteristics of the transistors with different gate voltage (V_G_) sweep ranges from ±20 V to ±100 V with a step of 20 V. The PDPPT-TT-based transistor exhibited ambipolar behavior at a drain voltage (V_D_) of −1 V. During the forward and reverse bias sweeps, the formation of hysteresis loop motivated us to explore the memory device applications based on the organic semiconductor. The hysteresis behavior of the OFETs could originate from the charge transport in the PDPPT-TT layer or the charge trapping at the interface of the PDPPT-TT layer and the ODTS/substrate. Although the memory hysteresis loop was not observable at the V_G_ sweep from +20 to −20 V (black symbols), the loop became more obvious as the V_G_ range increased from ±40 V (red symbols) to ±100 V (gray symbols). This trend of the hysteresis loop evolution with respect to the applied V_G_ range was summarized by plotting the threshold voltages (V_th_) of each forward and reverse sweep direction; the memory window changes are determined by the V_th_ shift between the forward and reverse sweeps (Figure 2c). As described in the transfer characteristics, the memory windows described by the V_th_ difference between the forward and reverse sweeps were negligible with the sweep voltage range (|V|) of 20 V. However, the memory windows were clearly defined as the sweep voltage range increased from 40 to 100 V. By applying the |V| of 100 V, the retention time was evaluated to characterize the operation stability. As shown in Figure 2d, however, the memory performance of this device architecture was not ideal due to the shallow trap-induced charge leakage, resulting in the short retention time.

### 3.2. Non-Volatile Memory Behavior of PDPPT-TT-Based Device

**Figure 3:**
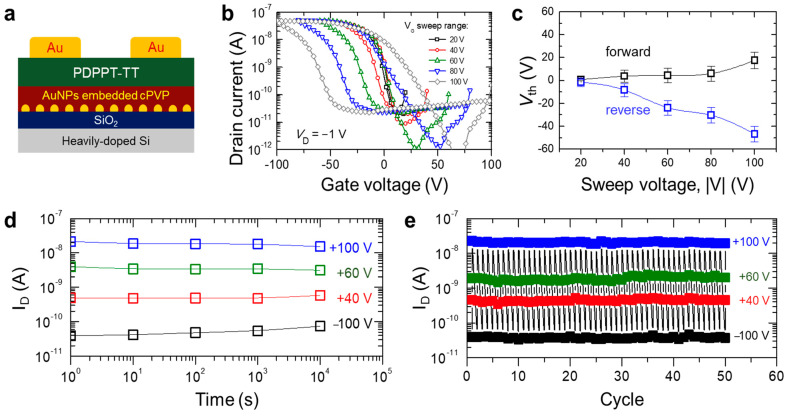
(**a**) Schematic of a non-volatile memory device by incorporating the AuNP-embedded c-PVP layer; (**b**) transfer characteristics of the memory device with respect to the gate voltage sweep range; (**c**) summarized plot of the memory windows defined as the threshold voltages of forward and reverse biases; (**d**) retention time of the device with different applied gate voltages; (**e**) cycling test of the device (the read voltage as V_G_ = 0 V).

To demonstrate the organic semiconductor PDPPT-TT-based non-volatile memory behavior, a AuNP-embedded c-PVP layer was incorporated as a charge trapping layer in between the PDPPT-TT layer and the SiO_2_ layer, as illustrated in Figure 3a. It is noted that the ODTS layer was not used for the non-volatile memory device applications due to the shallow trap which induced a relatively short retention time while the memory windows exceeded ~150 V (Figure S1). The transfer characteristics of the AuNP-incorporated PDPPT-TT device exhibited more obvious formation of the memory hysteresis loop (Figure 3b) as the V_G_ sweep range increased from 20 V to 100 V than those of device without the AuNP-embedded c-PVP layer. In comparison with the ambipolar transfer characteristics of the PDPPT-TT with a hole mobility of 0.037 cm^2^/Vs (Figure 2b), the AuNP-incorporated PDPPT-TT device showed a lower hole mobility of 0.023 cm^2^/Vs and negligible electron contributions (i.e., unipolar p-type transistor behavior). These mainly originated from an increased number of electron trap sites when the AuNP-embedded c-PVP layer was incorporated. Figure 3c exhibits the V_th_ of the forward and reverse biases with respect to the V_G_ sweep voltage range. In the range of ±20 V, the V_th_ difference, or the memory window, was almost negligible, similar to that of the device without the AuNP-embedded c-PVP layer. As the sweep voltage range increased to ±100 V, the V_th_ difference became more obvious up to ~70 V. To characterize the operational stability of the memory device, the retention and cycling tests were performed as shown in Figure 3d,e, respectively. In Figure 3d, the retention time was plotted after the various V_G_ was applied as 100 V (blue symbol), 60 V (green symbol), 40 V (red symbol), and −100 V (black symbol). Regardless of the applied V_G_, the retention time exceeded 10^4^ s after the V_G_ was applied. As shown in Figure 3e, a cycling test was performed to demonstrate the operational stability of the programmed and erased states while the V_G_ was applied repeatedly with a switching speed of 1 s. Under the application of arbitrary V_G_, the states were maintained after at least 50 cycles. These results indicate that the AuNP-embedded c-PVP layer-incorporated PDPPT-TT-based devices have non-volatile memory performances with good operational stability and reversibility. To further elucidate the role of AuNPs, a control device geometry was fabricated, as shown in Figure S2. This geometry exhibits that the retention time is very short although the memory window was as large as ~150 V.

## 4. Conclusions

In this work, we have demonstrated successful synthesis of an organic semiconductor PDPPT-TT for non-volatile memory device applications. The electrical properties of the semiconducting PDPPT-TT were evaluated by fabricating field-effect transistors. It showed ambipolar transfer characteristics and increased hysteresis loop windows as the gate voltage sweep range increased. However, the retention time of the programmed and erased states was not ideal for the non-volatile memory device applications. To solve the issue, a AuNP-embedded c-PVP layer was incorporated as an efficient charge trapping layer in between an SiO_2_/Si substrate and the semiconducting PDPPT-TT layer. The AuNP-embedded c-PVP layer-incorporated device showed unipolar transfer characteristics with negligible electron contributions due to a relatively larger number of the electron trapping sites; hence, the device exhibited an obvious memory window of ~70 V and the retention time of both programmed and erased states exceeded 10^4^ s with high repeatability once the controlled gate voltage was applied. This work provides a solution-processable organic semiconductor-based electrical device applications including ambipolar transistors and non-volatile memory devices.

## Supplementary Materials

The following are available online at https://www.mdpi.com/2072-666X/12/3/301/s1, Figure S1: electrical characteristics of the device with the incorporation of ODTS and AuNP-embedded c-PVP layers, Figure S2: electrical properties of the device with the identical structure without AuNPs.
