# Supplementary material for: Hysteresis Behavior of the Donor–Acceptor-Type Ambipolar Semiconductor for Non-Volatile Memory Applications
Source: Micromachines (Basel). 2021 Mar 12;12(3):301. doi: 10.3390/mi12030301 (PMC8000839; doi:10.3390/mi12030301)
Supplement: Supplementary file 1 [file micromachines-12-00301-s001.pdf]

Supplementary Materials

Hysteresis behavior of the donor-acceptor type ambipolar semiconductor for non-volatile memory applications

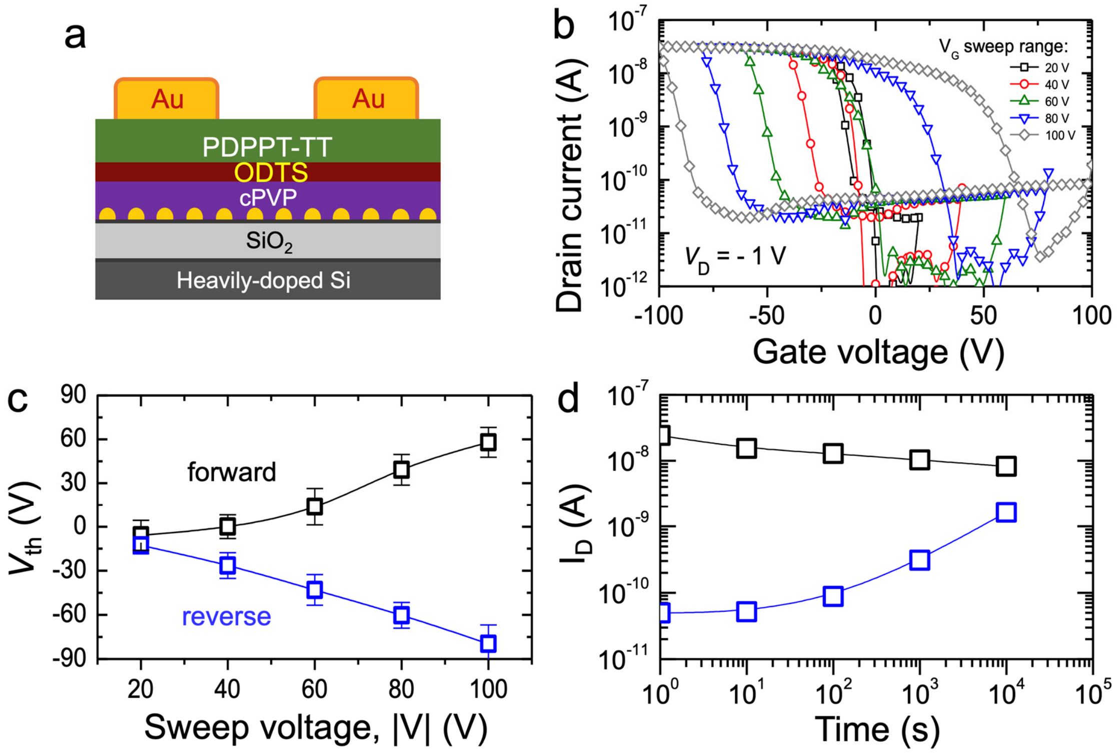

Figure S1. (a) Schematic of a device by incorporating ODTS and AuNPs embedded cPVP layers; **(b)** transfer characteristics of the memory device with respect to the gate voltage sweep range; **(c)** summarized plot of the memory windows defined as the threshold voltages of forward and reverse biases; **(d)** retention time of the device.

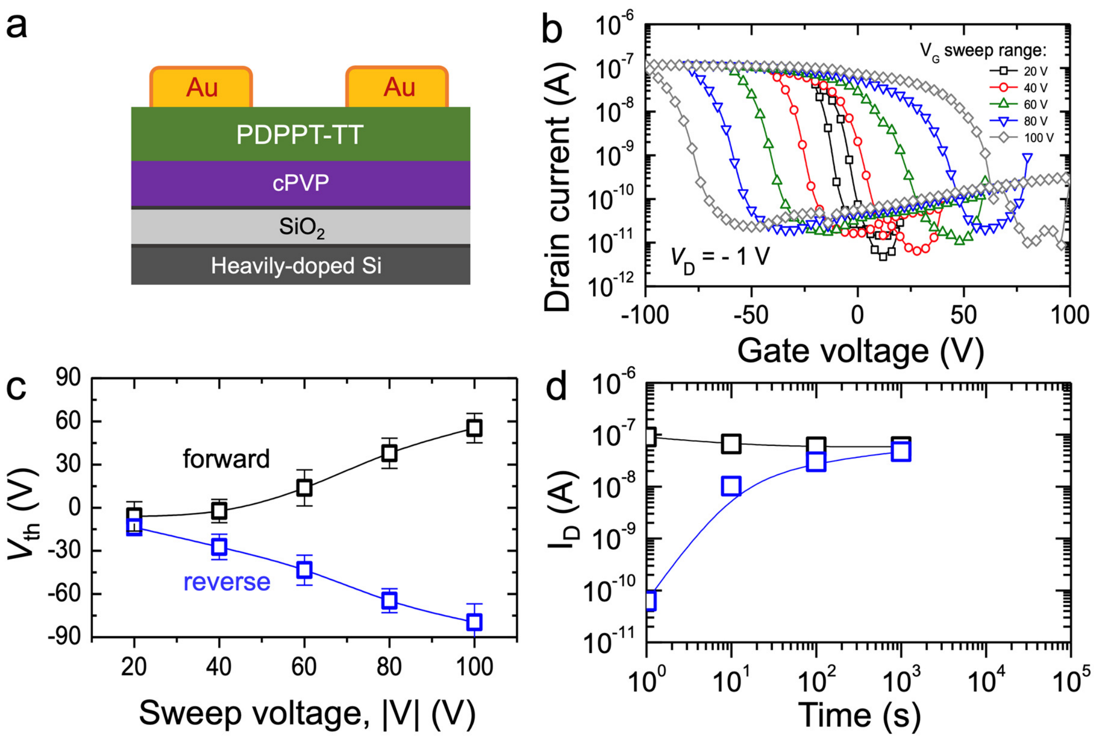

Figure S2. (a) Schematic of a device by incorporating a cPVP layer without AuNPs; **(b)** transfer characteristics of the memory device with respect to the gate voltage sweep range; **(c)** summarized plot of the memory windows defined as the threshold voltages of forward and reverse biases; **(d)** retention time of the device.
